# Managing Sleep in Adults with ADHD: From Science to Pragmatic Approaches

**DOI:** 10.3390/brainsci11101361

**Published:** 2021-10-16

**Authors:** Craig B. H. Surman, Daniel M. Walsh

**Affiliations:** 1 Clinical and Research Program in ADHD and Related Disorders, Massachusetts General Hospital, Boston, MA 02114, USA; dwalsh20@mgh.harvard.edu; 2 Harvard Medical School, Boston, MA 02215, USA

**Keywords:** attention-deficit hyperactivity disorder, sleep, treatment

## Abstract

Background: Sleep disorders and sleep problems commonly occur in adults with ADHD and add to functional impairment. Evidence-based treatments for sleep could improve function in the adult ADHD population. Methods: A literature review was conducted to present the clinical science informing treatment of sleep in adults with ADHD. Results: Six systematic prospective studies of sleep intervention in adults with ADHD were identified. Three of these, all including well-characterized ADHD patients, offered evidence for a significant effect of morning light therapy. Across the studies, preliminary evidence for melatonin, behavioral therapy, and weighted blankets were also found. Implication: Low-risk interventions such as light therapy may improve sleep in adults with ADHD, but many sleep interventions currently in use remain unstudied in the ADHD population. Considerations for evidence-informed practice and future research directions are discussed.

## 1. Introduction

Attention-deficit hyperactivity disorder (ADHD) is a neurobiological disorder associated with high levels of impairment in adulthood [1,2,3] and is estimated to affect up to 5% of adults worldwide [4,5,6]. There is strong evidence that adults with ADHD have an elevated risk for sleep-related problems, from surveys relying on screeners to identify ADHD [4,7,8], and clinical research studies that more completely characterized presence of ADHD [9,10,11,12,13,14]. For example, the study reported by the first author [14] compared 182 adults with ADHD to 117 adults without ADHD, finding that they went to bed 39 min later on average, were more likely to take an hour to fall asleep (17% vs. 4%), and were more likely to report daytime sleepiness (47% vs. 30%).

One influential early conceptualization of the ADHD phenotype, in the Diagnostic and Statistical Manual of Mental Disorders (DSM), Third Edition, even considered restless sleep to be a core trait of its child-focused definition of ADHD [15]. Current diagnostic criteria for ADHD require the presence of inattentive or impulsive-hyperactive traits. These traits must start in childhood, often persist into adulthood, and must impair function in two or more life settings [16]. By adulthood, the inattentive manifestation of ADHD is more common than impulsive-hyperactive presentations [3]. While sleep problems are not central to the DSM 5 diagnosis of ADHD, the central symptoms of inefficiency getting to, sticking with, and completing required daily tasks could result in unconventional or inadequate sleep schedules.

There could also be neurobiological differences that are common among individuals with ADHD that explain their high frequency of sleep problems. Emerging literature suggests that chronotype—the pattern of or preference for timing of sleep and wakefulness—may commonly be shifted later in adults with ADHD [8,17,18]. Of note, this was seen in studies conducted in unmedicated populations [8], suggesting that this shift is not due to the wake-modulating effects of sympathomimetic treatments used in treating ADHD. Some literature even suggests that genes associated with circadian patterns may be expressed differently at a genetic level in individuals with ADHD [18]. It is unclear, however, to what extent chronotype shifts are independent biological traits in these patients, or a result of ADHD behavior patterns that reinforce these chronotypes.

Inadequate sleep quantity and non-refreshing sleep patterns could compound the cognitive problems seen in adults with ADHD. Sleep deprivation is directly impairs cognition [19], but disruption of the pattern of sleep stages, and a resulting vulnerability to microsleep during the day, has also been identified as a cause of impaired cognition [20]. Sleep disordered breathing (abnormal levels of hypopnea and/or apnea) is a source of sleep pattern disruption [10]. Sleep disordered breathing may be highly prevalent in samples of adults with ADHD [21]. A review of six case–control studies of performance on cognitive measures in sleep disordered breathing and sleep apnea hypopnea syndrome, for example [22], found that cognitive deficit severity correlated with the degree of breathing disruption. Mild cases may be associated with mild attention and executive dysfunction, while more severe cases are associated with memory deficits. Further meta-analyses have also found consistent deficits in motor coordination, vigilance and executive function in sleep apnea hypopnea syndrome [23,24]. Treating sleep disordered breathing, and any other source of sleep pattern disruption, might thus reverse a source of cognitive impairment.

Adults with ADHD have high rates of neuropsychiatric comorbidity such as depression and anxiety disorders [3]. Because sleep problems are features of these comorbid conditions, they might be a source of the apparent association between ADHD and sleep problems. We are aware of only one study that attempted to disentangle the connection between ADHD and sleep problems in an epidemiological study. Analysis of National Comorbidity Survey-Replication study data found that adults who were likely to have ADHD had greater odds of four kinds of sleep problems. Any of these sleep problems occurred in 7.8% of those adults, with an odds ratio (OR) of 4.3. By problem, the study found: difficulty initiating: 10.1%, OR 3.8; difficulty maintaining: 10.3%, OR 3.8; early morning awakening: 12.6%, OR 5.3; and not feeling rested after ample time in bed: 8.6%, OR 4.2. This study, however, found similar magnitude odds of these problems occurring with any of the mental health comorbidities surveyed. Of the four categories, non-refreshing sleep was the most strongly associated with another measure of functional impairment [10].

Three studies have confirmed that elevated rates of self-reported sleep problems occur in adults with ADHD in the absence of comorbid mental health conditions [11,12,13]. For example, in the study mentioned previously that was conducted by our research group, sleep-related impairments survived analyses accounting for comorbidity [21]. In addition to the findings emphasized above, we also found that ADHD subjects, compared to controls, reported significantly more kinds of sleep problems, had a wider range of bedtimes, and were more likely to have difficulty going to bed, sleeping restfully, or waking in the morning. Adults with ADHD also experienced daytime sleepiness more often (OR = 2.23, *p* = 0.003). Of note, a survey presents evidence that daytime sleepiness may contribute to driving accidents in adults with ADHD [25]. Evidence that sleep is disturbed independent of comorbidity also comes from smaller studies that used objective measurement, such as one [26] that controlled for anxiety and depression comorbidity, confirming that sleep onset latency and sleep efficiency problems were associated with ADHD.

To understand the evidence basis for treatment of sleep problems and disorders in adults with ADHD, we conducted a systematic search for prospective studies of sleep interventions. Further, we explored the forms of evidence-based treatment for sleep disorders in general, and their applicability to adults with ADHD.

## 2. Materials and Methods

**Figure 1:**
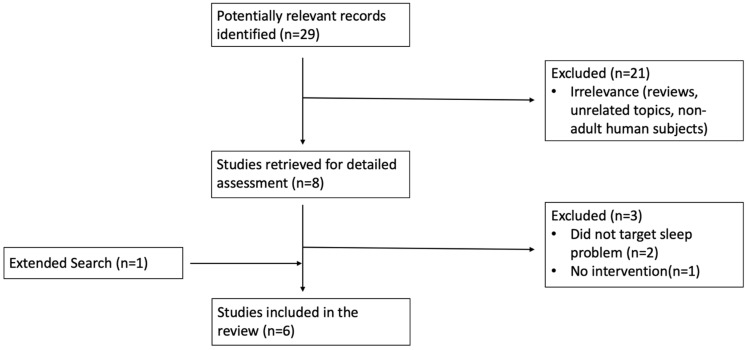
Flowchart of Literature Search using PubMed.

As can be seen in the following flowchart (Figure 1), we conducted a literature search in PubMed, exploring the terms “ADHD,” “sleep,” and “treatment,” excluding studies of children. In addition, we supplemented our report with one study that the authors were aware of and that was published in a peer-reviewed journal. Studies were included that were designed to evaluate the effect of interventions targeting sleep problems in populations of adults with ADHD that included systematic measurement of sleep.

## 3. Results

As can be seen in Table 1, we found six studies that explored a range of interventions—three of them studied bright light therapy [27,28,29], one studied a behavioral treatment [30], one studied weighted blankets [31], and one studied ramelteon [32]. Only the largest bright light study [27] (which also involved melatonin treatment) and the weighted blanket study made comparisons between an intervention and a control condition. All of the bright light studies used methods sufficient to suggest that they included individuals with a DSM IV or DSM 5 diagnosis of ADHD. The behavioral therapy [30] and weighted blanket [31] studies did not systematically confirm a full ADHD diagnosis; instead, they applied their interventions in a population that included individuals with prior ADHD diagnosis. While each study involved behavioral change, Jernelov et al. [30] specifically studied behavioral intervention to support sleep hygiene, educating individuals on management of light exposure and stimulant medication timing.

All of the studies reported favorable effects on sleep pattern, although varied measures were used across studies. Two of the bright light studies demonstrated a significant shift in dim light melatonin onset, measured via saliva sampling. The rise of melatonin is an indicator of circadian timing [27,28].

It is notable that through extended searches beyond our core search, we were only able to identify one controlled study of a pharmaceutical intervention in adults with ADHD targeting sleep. This study found mixed impact of ramelteon on sleep-related measures vs. placebo, with earlier mid-sleep time but also worse sleep fragmentation and increased daytime fatigue [32]. Of note, a small chart review of 6 cases suggested that 30 mg mirtazapine, which has antihistamine and 5ht1 blocking effects, improved stimulant-associated insomnia in adults with ADHD without systematic sleep measures [33].

## 4. Discussion

Our review found evidence that bright light therapy may be a viable intervention to shift sleep patterns and improve functioning in adults with ADHD. This evidence was generated across three studies including a total of 96 adults with ADHD. To our knowledge, no other interventions for sleep in adults with ADHD have been evaluated in replicated studies. Below, we further explore factors unique to ADHD populations and principles of sleep management that clinicians may consider when addressing sleep in adult ADHD populations.

### 4.1. Approaches for Assessing Sleep Problems and Disorders in Adults with ADHD

All adults with possible ADHD should be evaluated for sleep problems as part of a comprehensive differential assessment. Interviewing should identify sleep problems that are a product of behaviors related to under-treated ADHD, or due to comorbid sleep disorders. To identify the etiology of sleep problems, it is useful to map the patient’s history of extremes in sleep quantity, sleep quality, or non-refreshing sleep. In this exploration, it can be clinically useful to separate chronic and episodic patterns.

Important examples of chronic sleep problems to identify include longstanding “night owl” behavior that can reflect shifted chronotype, or napping that can reflect narcolepsy or idiopathic hypersomnia. Examples of intermittent sleep problems and their sources include insomnia during a personal crisis, sleep pattern changes unique to perimenopausal hormonal changes, or exacerbation of apnea with increase in body weight. Screening questions may identify other sleep-wake disorders, of which DSM 5 identifies 11 categories [16], but diagnosis may require objective measurement of sleep characteristics. Formal sleep study measurement, for example, can clarify diagnoses of disordered breathing/apnea or narcolepsy.

Asking about history and pattern of sleep problems can also facilitate identification of comorbid neuropsychiatric conditions other than sleep or ADHD. Substance use disorders are common among individuals with ADHD, and could contribute to sleep-wake disturbances. Clinicians have come to associate particular patterns with other comorbidities—for example, several-day episodes of reduced need for sleep are common in bipolar disorder, trouble falling and staying asleep is common with flares of anxiety disorders, nightmares are a feature of PTSD and anxiety disorders, early morning awakening is common in depression and adjustment disorders, and elongated sleep times are common in other forms of depression as well as seasonal affective presentations.

Assessment should also identify sleep-promoting and disrupting agents that patients are taking. Stimulants such as methylphenidate and amphetamine salts that treat ADHD have well-known wake-promoting effects. While atomoxetine, a non-stimulant used for ADHD, does not have strong wake-promoting effects, some patients report mid-sleep awakening on atomoxetine, and that changing the time they take it can eliminate that phenomenon. Focusing on what people consume, from food to caffeine to alcohol, and when, can also provide opportunities for education on sleep-healthy habits.

The dose timing, elimination half-life, and mechanism of delivery of a particular pharmaceutical may influences the likelihood it will wear off in time to avoid sleep initiation problems. However, analyses of Pittsburgh Sleep Quality Index clinical trial data for 12–16 h long-acting stimulants suggests that, on average, even these long-duration agents can be tolerated without shifting individuals from good to bad, or from bad to good sleep [34,35]. A recent systematic assessment of a large clinical population of adults with ADHD even found that stable ADHD treatment was associated with lower rates of insomnia disorder than untreated ADHD [36].

Clearly, a full evaluation of sleep requires gathering a significant amount of information. Clinicians may be unsuccessful at collecting data from adults with ADHD if it depends on “homework”, because ADHD and associated executive function challenges limit follow-through on such self-directed tasks. For example, clinicians should not expect untreated adults with ADHD to reliably fill out serial sleep diaries. In fact, the authors of one of the light therapy studies we reviewed noted “while we hoped to assess (light therapy) compliance and sleep times using daily logs, the core deficits of these subjects often interfered with this very task [29].” Sleep diaries are a staple of insomnia diagnosis and sleep hygiene improvement, but it may be more practical to use a brief retrospective sleep diary, such as the retrospective self-report version of the Consensus Sleep Diary [37].

Evaluation of sleep health needs to go beyond asking individuals if they have sufficient duration of sleep or if they are tired during the day. Individuals may not have either of these problems, but still have significant compromise of their sleep pattern. Clinicians should inquire about phenomena such as napping, gasping, snoring, excessive sleep movement, and parasomnias. Validated screeners for sleep disorders are available, but clinicians may find it possible to distill the essential concerns they address into a screening interview that covers presence of daytime fatigue [38] preferred wake and sleep patterns [39], alignment between native alertness and the 24 h clock [40], and traits that predictive likelihood of sleep apnea [41]. Efforts have been made to develop short screening questions for some sleep-wake disorders. For example, this question had 100% sensitivity and 96.8% specificity for restless leg syndrome in a neurology outpatient population: “When you try to relax in the evening or sleep at night, do you ever have unpleasant, restless feelings in your legs that can be relieved by walking or movement?” [42]. Similarly, asking individuals their “energy level” in the morning and the evening may efficiently screen for preferred sleep and wake patterns [43].

Measurement of sleep by self-report and by objective study can produce divergent findings in the same individual. There is evidence that actigraphy offers a closer approximation of sleep duration as measured by polysomnography than sleep surveys do [44]. One study in adults with ADHD provides an example of this divergence between subjective and objective measures. Philipsen et al. found that among 20 adults with ADHD compared to 20 matched controls [12], self-reported sleep time, quality and efficiency were lower, and correlated with periodic limb movement frequency, but not with polysomnographic measures of sleep efficiency, length, or onset latency [12].

Personal phones and wearable devices have become a common means of sleep data collection, through a wide array of applications that collect patterns of user device interation and motion. While it is likely variable how well such measurements serve as a proxy for actual actigraphy or sleep laboratory evaluation, they may serve as a practical means of collaborating with patients on sleep monitoring. Pre-programmed personalized messages are another method that can be used to prompt patients to collect data [45], and could also be used to prompt and measure sleep habit changes. Further research may clarify that particular sleep assessment methods are most likely to lead to treatment interventions in ADHD populations. For example, our review suggests that circadian rhythm measurement may translate into actual improvement in daytime function through deployment of morning light therapy.

### 4.2. Approaches to Treating Sleep Disorders in ADHD

Helping people sleep better involves asking them to change their behavior, whether it involves devices, medications, or adopting new habits. As we have emphasized already, adults with ADHD often struggle to follow through on their plans, and thus may struggle to change behaviors designed to help them sleep better. Recommendations should be implemented in the context of accommodations for the planning, task-switching, and organizing challenges that are often seen beyond the core traits of ADHD [3,46].

In practice, the first author finds it helpful to understand the patient’s motivation to get better sleep. Next, it is practical to understand if the patient has had adequate and refreshing sleep patterns in the past, and what change in habits or environmental circumstances has occurred since. This may help patients focus in on moments during the day or evening that they can make sleep-promoting choices, and what environmental changes may be most conducive to maintaining healthy sleep patterns [47]. Accommodations may be important to assist adults with ADHD in such behavior change. For example, Jernelov et al. [30] incorporated elements of CBT developed for ADHD by Safren et al. [48] in their adaptation of CBT for insomnia, such as use of calendars and alarms. These simple tools can help patients map out, and remember, the critical moments they need to facilitate their future sleep behavior [48].

When patients have comorbid conditions, prioritizing what to treat first can be challenging. It is generally accepted that the most biologically or functionally compromising primary condition should be treated first. In many cases, treatment of ADHD, or even assessment of its presence, should wait until a major sleep disorder or another major condition is first managed. In contrast, where ADHD symptoms or a comorbid mental disorder are the source of sleep problems, treating these primary conditions can eliminate the need for additional sleep support.

Biological and environmental disease-modifying factors can change, the patient’s area of concern may shift, and treatments themselves may cause additional impairment. Stimulants, for example, may hide the sedative hangover effects of sleep agents taken the night before. Because treatment priorities can change over time, the first author has found it very useful to establish what measures will be followed for each syndrome identified prior to initiating treatment, and to re-evaluate baseline symptoms off treatment intermittently.

While there is no replicated literature to guide choice of sleep treatments in adults with ADHD specifically beyond light therapy, there are evidence-based interventions for sleep disorders. These include treatments that we can say are likely to have more effect that placebo. Cognitive behavioral treatments for sleep (which were utilized in the study by Jernelov et al. discussed in our review [30]) utilize stimulus control and sleep restriction, and replacement of negative mindsets about sleep with more positive expectations. Such interventions appear to have medium effect sizes on sleep, and may be as effective as medications with sedative effects for insomnia [49,50,51].

It is prudent to educate patients about, and prescribe, approaches that may have the least impact on daytime function. For example, the results of the one controlled medication treatment study we found that included adults with ADHD are cautionary: the novel melatonin agonist ramelteon was associated with an advance in dim light melatonin onset, but also an increase in daytime sleepiness [32]. While drug half-lives and clinical trials of some agents suggest that they may target initial, middle, or late sleep impairments, it is the first author’s experience that the duration and quality of sedation effect among these agents varies significantly from patient to patient.

When insufficient sleep duration is not responsive to behavioral or light therapy, drug and supplement treatments may be more appropriate. Several agents are approved for insomnia management over the short term. One systematic review emphasized that clinicians have littled evidence-basis to rely on in their selection of agents for *chronic* insomnia, rating every product reviewed as having weak levels of evidence [52]. When recommending psychoactive agents for insomnia, it seems health-promoting to consider non-habit forming, and daytime-function sparingagents first where effects are otherwise equal. Opinion statements [53] have suggested that melatonin may be favorable as a first choice supplement for shifting sleep time, using lower doses 4–6 h before bedtime to establish biological evening, and/or higher doses before bedtime for sedative initiation of sleep.

In many comorbid presentations, clinicians will target sleep problems while addressing another non-ADHD syndromes. Ideally, there would be systematic study of sleep effects for such dual-purpose treatments. Interestingly, although amitriptyline is widely used for its sedative properties in neurological populations, a recent review could not find strong evidence for its impact on sleep [54]. This same review evaluated evidence for sleep benefit from other antidepressants, finding that only doxepin and trazodone had the highest scientific evidence among these agents, based on self-report evidence for sleep improvement. A recent study [55] considering effects of trazodone on cognition measures across 16 studies with at least 25 mg exposure for one week suggested mixed evidence for a dose-dependent negative effect on next-day cognition. These findings, again, emphasize the important trade-off that clinicians may face in trying to optimize sleep while supporting daytime function.

## 5. Conclusions

Our review found promising evidence that morning bright light therapy may improve sleep-related problems and even daytime function in adults with ADHD. This may be due to common occurrence of delayed sleep chronotype. Clinicians should systematically screen for sleep problems and disorders in the adult ADHD population. Further research may clarify which subjective and objective assessment methods are most efficient. Cognitive behavioral strategies and environmental accommodations may promote adoption of better sleep hygiene habits and sleep–wake cycles, with low risk. Pharmacotherapies and non-prescription agents used in adult ADHD populations should be chosen to minimize impact on sleep, and maximize daytime function. Clinicians may find that systematic, interactive, and structured serial assessments will provide accountability for the patient and facilitate sleep management in adults with ADHD.

## Figures and Tables

**Table 1:** Prospective studies of sleep intervention in adults with ADHD.

Study (Year)	Population	Comorbidity and Other Treatments	Intervention	Design	Baseline Characterization Related to Sleep	Significant Findings
Van Andel et al. (2021) [27]	*N* = 51 DSM-IV ADHD and Delayed Sleep Phase Syndrome, (m = 29.53 yrs) (32 females)	Mental health comorbidity exclusionary, no ADHD supports reported	Advancing melatonin (0.5 mg/d) timed to dim light melatonin onset (DLMO) with and without bright light therapy (BLT), 3 weeks	Three-arm randomized placebo controlled	DLMO, Sleep Diagnosis List (SDL), Sleep Hygiene Questionnaire (VSH)	Melatonin advanced DLMO by 1 h and 28 min, *p* = 0.001. Melatonin plus BLT advanced DLMO by 1 h and 58, *p* < 0.001. Placebo had no effect on DLMO. Melatonin reduced ADHD symptoms by 14% *p* = 0.038, which returned to baseline 2 weeks post-treatment.
Fargason et al. (2017) [28]	*N* = 16 DSM-IV ADHD (m = 35.25 yrs) (9 females)	Mental health comorbidity exclusionary. 11 on amphetamine drugs, 1 on buproprion	BLT, 2 weeks after 1 week baseline	Open treatment	DLMO PSQI Sleep Diary	BLT advanced DLMO by 31 min *p* = 0.002. vs. 1 week baseline BLT advanced mid-sleep time by 57 min *p* = 0.004. “Sleepiness” ratings in sleep diary were reduced *p* = 0.033. PSQI overall quality score improved *p* < 0.001.
Jernelov et al. (2019) [30]	*N* = 19 people with clinical record of ADHD and self reported sleep problems (m = 37) (13 females)	Participants had an average of 3 mental health conditions in the last year. 11 on forms of amphetamine, 7 on methylphenidate, 8 on current sleep agents	Group sessions of CBT-i for insomnia, 10 weeks	Open treatment with three-month follow up	SLEEP-50 Karolinska Sleep Questionnaire Insomnia Severity Index	Insomnia severity decreased *p* = 0.002. At three-month follow up, insomnia severity had further improved *p* < 0.0001 from pre-treatment.
Rybak et al. (2006) [29]	*N* = 29 DSM ADHD by Conner’s and Wender Utah Scales (14 females)	41% had major depression, 13% had seasonal affective disorder; 7 subjects taking psychostimulants only, 4 taking antidepressants only, 4 taking both	BLT, 3 weeks after 1 week baseline	Open treatment	Brown Adult ADD Scale Conners’ Adult ADD Scale Horne–Ostberg Morningness-Eveningness Questionnaire Neuropsychological Tests	Significant phase advance in circadian preference, *p* = 0.016, decrease in self-report ADHD symptoms (*p* = 0.001), and change in 10 out of 18 neuropsychological tests (*p* range = 0.05 to 0.001).
Ekholm et al. (2020) [31]	*N* = 13 cohort subset with clinical record of sleep disorders and ADHD	Various concurrent treatments	Weighted chain blankets, 4 weeks	Between group (vs. plastic chain blanket)	Insomnia severity index, fatigue symptom inventory, hospital anxiety and depression scale, wrist actigraphy	Insomnia severity improved more than in light blanket group *p* = 0.003.
Fargason et al. (2011) [32]	*N* = 36 adults (18 females) with DSM-IV ADHD	Individuals with elevated Hamilton Anxiety/Depression ratings or other cause of insomnia excluded.	Ramelteon 8 mg, 2 weeks each of placebo, washout, and active, after 1 week baseline	Open treatment crossover	Actigraphy, Epworth Sleepiness Scale (ESS), and ADHD-RS	7.0 ± 32.3 min phase advance; placebo 39.2 ± 44.6 min phase delay (*p* = 0.046 for both); # participants with significant ESS score more than doubled *p* < 0.017.
